# Copland’s Dictionary of Practical Medicine, Part XX

**Published:** 1848-12

**Authors:** 


*Copland's Dictionary of Practical Medicine, Part XX.*—This comprehensive and erudite work is slowly advancing toward completion. Part twentieth has been recently issued, embracing *Poisons* and *Pregnancy.* There appears to be no flagging, either on the part of the Author or American Editor. This is all that need be said by way of encomium, for the profession are sufficiently aware of the merits of the early numbers. When finished it will constitute in itself a medical library. As a work of reference it will be immensely valuable.

